# Role of SNAREs in Neurodegenerative Diseases

**DOI:** 10.3390/cells10050991

**Published:** 2021-04-23

**Authors:** Azzurra Margiotta

**Affiliations:** 1 Department of Biology, Faculty of Medicine, Masaryk University, 62500 Brno, Czech Republic; azzurramarg@libero.it; 2 International Clinical Research Center, St. Anne’s University Hospital, 65691 Brno, Czech Republic

**Keywords:** SNAREs, neurodegenerative disease, ALS, Parkinson’s disease, Alzheimer’s disease, VAMP2, syn1, SNAP-25

## Abstract

Neurodegenerative diseases are pathologies of the central and peripheral nervous systems characterized by loss of brain functions and problems in movement which occur due to the slow and progressive degeneration of cellular elements. Several neurodegenerative diseases are known such as Alzheimer’s disease, Parkinson’s disease and amyotrophic lateral sclerosis and many studies on the molecular mechanisms underlying these pathologies have been conducted. Altered functions of some key proteins and the presence of intraneuronal aggregates have been identified as responsible for the development of the diseases. Interestingly, the formation of the SNARE complex has been discovered to be fundamental for vesicle fusion, vesicle recycling and neurotransmitter release. Indeed, inhibition of the formation of the SNARE complex, defects in the SNARE-dependent exocytosis and altered regulation of SNARE-mediated vesicle fusion have been associated with neurodegeneration. In this review, the biological aspects of neurodegenerative diseases and the role of SNARE proteins in relation to the onset of these pathologies are described.

## 1. Introduction

Neurodegenerative diseases are a group of pathologies affecting the central and peripheral nervous system, they are characterized by a variety of clinical and pathological expressions and occur due to the loss of neurons in different regions [1,2]. Neurodegenerative disorders of the central nervous system (CNS) relate to diseases in the cerebral cortex, the basal ganglia, the brainstem, the cerebellum and the spinal cord [3]. Alzheimer’s disease (AD) is the most common type of dementia affecting the cerebral cortex [3], while Parkinson’s disease (PD) is the second most common neurodegenerative disease and involves the dopaminergic neurons in the substantia nigra pars compacta (SNpc). Huntington’s disease (HD) is an adult-onset neurodegenerative disorder affecting the structure and functions of the striatum, cerebral cortex and hippocampus and it is characterized by chorea and dementia [4,5]. Another disease of the CNS is multiple sclerosis (MS), which is a chronic inflammatory disease [6]. Neurodegenerative diseases of motor neurons (MNDs) are a spectrum of neurodegenerative disorders that progressively affect motor neurons in the anterior horn of the spinal cord, brainstem, cortex and pyramidal tract, determining the death of the upper motor neuron (UMN) in the motor cortex and/or of the lower motor neuron (LMN) in the brain stem cells and spinal cord [7]. MNDs are clinically classified in subgroups in relation to which motor neurons are involved. Amyotrophic lateral sclerosis (ALS) is characterized by degeneration of both upper and lower motor neurons and it is sometimes reported as MND since ALS is the most common form [8]. Among MNDs that affect only lower motor neurons progressive muscular atrophy (PMA), progressive bulbar palsy (PBP), spinal muscular atrophy (SMA) and post-polio syndrome are included. On the contrary, primary lateral sclerosis (PLS) regards the alteration of the upper motor neurons [8]. MNDs lead to early death and are characterized by progressive muscle weakness, wasting and cramps if the UMNs are affected and brisk reflexes and functional limits when UMNs are lost [7]. Moreover, neurodegenerative diseases can be genetic or sporadic which occurs in the majority of the cases [3].

The classification of neurodegenerative disorders is challenging due to the high number of pathologies (a few hundred) and difficulty in making clear clinical and pathological distinctions [3]. Several molecular pathways are involved in neuronal survival and many important proteins are mutated or display an altered function in the different diseases [1,2]. Interestingly, cellular elements of the nervous system are affected by abnormal deposition of protein and alterations in the metabolic functions [9]. Therefore, the molecular classification of neurodegenerative disorders is based on these proteins and the most common diseases are amyloidopathies, tauopathies, synucleinopathies and transactivation response DNA binding protein 43 (TDP-43) proteinopathies [10]. A major role in the onset of neurodegenerative diseases is played by the loss of synapse integrity and function and this is considered an early event in many neurodegenerative disorders [11,12,13]. Moreover, a contribution of glial cells to human neurodegenerative diseases due to protein accumulations has been discovered [11,14,15,16,17,18,19,20,21]. Interestingly, soluble N-ethylmaleimide-sensitive factor attachment protein receptor (SNARE) proteins are important for neurotransmitter release and other functions in neurons and glial cells [22].

SNARE proteins are generally small proteins of around 100–300 aminoacids and they mediate the fusion of biological membranes by localizing both at the vesicular membrane and on the target membrane (for further details please refer to [23,24,25,26,27]. They share a common domain, the SNARE motif, which is normally of 60 aa in length. Most of them contain a C-terminal hydrophobic region which is necessary for anchoring to the membrane. They associate in complexes made either of three or four SNARE proteins, pull the lipid bilayers together and initiate membrane merging [28]. SNARE proteins present an α-helix domain, the SNARE domain, responsible for the SNARE binding and are classified in Q- or R-SNAREs in relation to the residue (glutamine or arginine, respectively) that is highly conserved in the hydrophilic layer in the center. Q-SNAREs are further subdivided into Q_a_, Q_b_, Q_c_ and Q_b-c_ SNAREs, depending on their location in the four helix-bundle and on how many SNARE domains they contribute with to the formation of the complex [28]. Normally, four SNAREs (three different Q-SNAREs and one R-SNARE) compose the complex driving membrane fusion, whereas Q_b-c_ SNAREs contribute with two SNARE motifs, therefore they take part in the ternary complexes together with one Q_a_ and one R-SNARE [28]. The SNARE complex is due to the fusion of two vesicles or a vesicle to the plasma membrane (PM) and the Q SNAREs are normally located at one compartment while the R SNARE on the other [28]. SNARE proteins have been involved in many fundamental neuronal functions such as neurite initiation and outgrowth, axon specification, axon extension, synaptogenesis and synaptic transmission [29]. The main SNARE complex that has been associated with exocytic fusion in neurons is the one composed of vesicle-associated membrane protein 2 (VAMP2)/synaptosomal-associated protein 25 (SNAP-25)/syntaxin-1 (syn1) [30]. The Sec1/Munc-18-like (SM) protein Munc18-1 regulates the formation of this SNARE complex [30].

VAMP2 (also known as synaptobrevin2, syb2) is an R-SNARE protein that is highly expressed in neurons [31,32,33] and localized on vesicles. It takes part in a SNARE complex together with the Q_b-c_ SNARE SNAP-25 and the Q_a_ SNARE syn1 which are present on the PM and it is important for the neurotransmitter release [22]. This process consists of several steps. At the presynaptic terminal during a nerve impulse the depolarization of the membrane (change in the electric charge distribution) occurs, the voltage-gated Ca^2+^ channels are activated, the ions enter the cells, the docking proteins change conformation and fusion of the vesicle with the PM with subsequent release of the neurotransmitter in the synaptic cleft occurs. In this context, VAMP2-positive vesicles reach the PM and, after depolarization of the synaptic site, calcium ions enter the cell and trigger the formation of the SNARE complex containing VAMP2/SNAP-25 and syn1. This SNARE complex is based on the alignment of the four helices with VAMP2 and syn1 contributing one SNARE domain each and the Q_b-c_ SNARE SNAP-25 contributes two. Subsequently, a conversion from trans- to cis-configuration (where the SNARE proteins reside in the same membrane) and the formation and expansion of the fusion pore occurs and the material contained in the vesicles is released [22]. The cis-SNARE complex is then disassembled through ATP hydrolysis by the N-ethylmaleimide sensitive factor (NSF), a cytoplasmic ATP-ase protein, and its soluble attachment proteins [22].

VAMP2 is involved both in synaptic vesicle exocytosis and endocytosis [34,35,36,37] and its loss in *Drosophila* led to neurodegeneration [38]. Furthermore, it is known that perturbations of its N-terminal domain inhibit neurotransmitter release [39].

Some information on the association between SNARE proteins and neurodegenerative diseases have been discovered recently and are reported in Table 1. However, the mechanism through which the impairment of the SNARE complex formation or functionality is related to neurodegeneration is still unclear. Molecules affected by SNARE proteins and their functions in neurodegenerative diseases are reported in Table 2. A role of SNARE proteins in the pathophysiology of AD, PD and ALS has been discovered [30,40,41,42,43,44,45,46,47,48].

## 2. AD

AD is a slowly progressive neurodegenerative disease characterized by the formation of neuritic plaques formed by amyloid beta (Aβ), neurofibrillary tangles (NFTs) composed of phosphorylated tau protein, loss of neurons in the hippocampus and dementia. AD has been discovered more than 100 years ago and since then several pathogenic mechanisms have been proposed and the main two hypotheses are the tau and the amyloid ones [73]. However, gamma oscillations, prion transmission, cerebral vasoconstriction, growth hormone secretagogue receptor 1α (GHSR1α)-mediated mechanism, and infection theories have been suggested (reviewed in [73]). This disease is caused by several molecular mechanisms, such as Aβ and tau aggregation, inflammation and oxidative damage [74,75]. The main cause of the onset and progression of AD is the hyperphosphorylation of the microtubule-binding protein tau and its aggregation. Tau is highly expressed in neurons where it localizes mainly in axons, promotes the self-association of tubulin into microtubules and interacts with several other molecules, regulating cytoplasmic transport and neuronal signaling. In AD-affected patients, tau is abnormally phosphorylated, interacts with other tau molecules and polymerizes to form insoluble paired helical filaments (PHFs) and straight filaments (SFs), leading to the formation of intraneuronal fibrillar deposits or NFTs [74,75]. NFTs decrease the number of synapses and determine neurotoxicity and neuronal dysfunction [73].

Neuritic plaques are constituted by the accumulation of Aβ due to the activity of the proteolytic cleavage enzymes on the Aβ precursor protein (APP) via the amyloidogenic pathway giving the final molecules Aβ40 and Aβ42 [74]. Interestingly, α-, β-, γ- and η-secretases can act on APP through three different pathways. The amyloidogenic pathway is based on the action of β- and γ-secretases producing C-terminal fragment (CTF)-β and different lengths of Aβ, where Aβ40 has strong neurotoxicity action and Aβ42 is associated with aggregation and plaque formation. The non-amyloidogenic pathological pathway is regulated by α- and γ-secretases, producing CTF-α, the soluble ectodomain of APP-α (sAPPα) and other smaller fragments which are neurotrophic and neuroprotective for nerve cells. Finally, η-secretase regulates the third pathway which is the alternative processing route under physiological conditions [73]. Aβ is a transmembrane protein and in AD is deposited in the hippocampus and in the basal segment, recruiting more Aβ, forming aggregates and inducing mitochondrial damage, unstable homeostasis and synaptic dysfunction [73]. Interestingly, in relation to the formation of neuritic plaques, microglia and astrocytes are activated and inflammatory reactions and oxidation occur. Finally, dysfunction of neuronal elements and apoptosis take place leading to AD [73].

**Figure 1:**
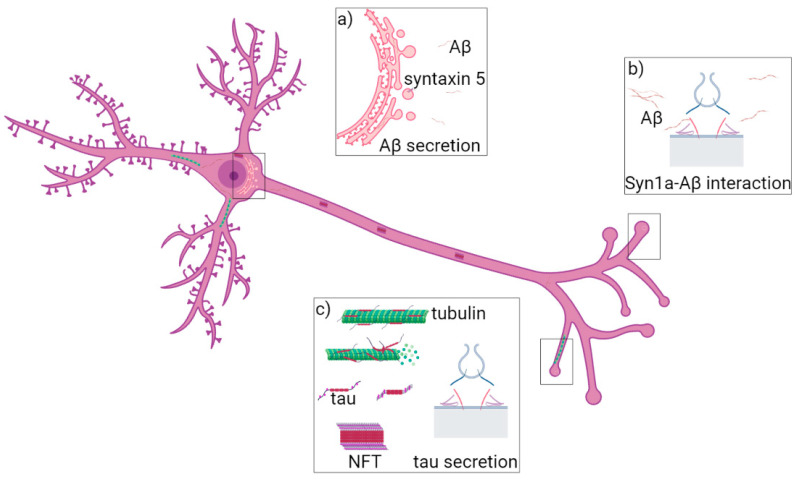
Role of syntaxin-5, syntaxin-1a and VAMP8 in AD. (**a**) Syntaxin-5 (violet) regulates membrane fusion at ER (pink) and it is responsible for Aβ42 peptide (yellow) accumulation. (**b**) Syntaxin-1a (red) interacts with Aβ monomers and oligomers (from yellow to brown), which block the SNARE complex formation and exocytosis. (**c**) VAMP8 (blue) is localized at vesicles fusing to the PM and regulates tau secretion.

AD can be classified as late-onset (LOAD), sporadic (SAD) or early-onset (EOAD) and familial (FAD). Mutations in the *APP*, presenilin-1 (*PSEN-1*), presenilin-2 (*PSEN-2*) and apolipoprotein E (*APOE*) are associated with EOAD [75]. Among the genes associated with AD, mutations in Clusterin gene (*CLU*) and Bridging Integrator 1 (*BIN1*) have been linked to LOAD [74]. The SNARE proteins syntaxin-5, syntaxin-1, SNAP-25, VAMP2 and VAMP8 have been found to have a role in AD onset (Figure 1) [40,42,44,49,50,51,52,56,60,61,76].

### 2.1. Syntaxin-5 Is Involved in Aβ42 Accumulation

Syntaxin-5 is a Q_a_ SNARE expressed ubiquitously and it has a relevant function in the endoplasmic reticulum (ER) as it regulates membrane fusion at ER and Golgi apparatus. Its overexpression has been linked to βAPP accumulation and reduced Aβ secretion [42,44]. Oxidative stress by ozone exposure determines the overproduction of Aβ42 peptide, its accumulation in the hippocampus and it causes neurodegeneration in the brain of rats [42]. Production and accumulation of Aβ42 occur in mitochondria, intracellular membranes and ER. In the latter case, expression of syntaxin-5 was increased and associated with neurodegeneration [42]. Similarly, ER stress signals determined the upregulation of syntaxin-5 and of the Q_c_ SNARE BET1 [44]. It has been discovered that the expression of syntaxin-5 upon ER-Golgi stress or apoptosis was necessary for neuron protection and participated in βAPP processing and suppression of amyloid-β peptide secretion [44]. In support of this, the knockdown of syntaxin-5 during apoptosis enhanced the vulnerability of neurons [44]. Interestingly, syntaxin-5 interacted also with presenilin holoproteins when the SNARE protein was localized at the ER or cis-golgi apparatus. Moreover, besides inducing an accumulation of presenilin holoproteins, syntaxin-5 overexpression determined an accumulation and reduced secretion of Aβ peptide also in COS-7 cells [56]. Indeed, the role of syntaxin-5 in the pathophysiology of AD is related to the accumulation and secretion of Aβ peptides produced via the amyloidogenic pathway [42,44]. Therefore, syntaxin-5 is considered a potential stress-responsive factor that regulates APP processing and neuronal survival [43].

### 2.2. SNAP-25, Syntaxin-1a and VAMP2 Role in Neurodegeneration

SNAP-25 is a membrane-associated protein that is mainly localized in the nerve terminals in the brain where it regulates the docking and/or fusion of synaptic vesicles to the PM by interacting with voltage-gated calcium channels. SNAP-25 is also important for synaptic vesicle recycling, neurite extension, neuron repair and synaptogenesis [60,76]. Interestingly, its presence in the cerebrospinal fluid (CSF) has been related to synaptic damage that precedes neuronal loss and impaired memory formation in AD [60]. In particular, it has been proven that the levels of SNAP-25 and of the SNAP-25/Aβ42 ratio were increased in patients with progressive mild cognitive impairment and AD compared to cognitively normal individuals and could be used as a diagnostic tool also to predict the conversion from progressive mild cognitive impairment to AD [60]. Interestingly, the levels of VAMP2, syntaxin-1a and SNAP-25 were reduced in human brain samples [40]. A role for SNAP-25 as a biomarker has been detected also by Agliardi and colleagues in neuron-derived exosomes where its abundance was reduced in AD patients [61].

Munc 18-1 is a SNARE-associated protein and it is essential for regulating the fusion of vesicles containing neurotransmitters. Interestingly, defects in the trafficking of the SNARE protein syntaxin1a in Munc 18-1 −/− neurons were involved in neurodegeneration with several pathological features of Alzheimer’s disease [49]. In fact, Munc 18-1 acted as a chaperone in order to stabilize syntaxin-1a and in Munc 18-1 −/− the SNARE protein displayed somatic accumulations, as well as the receptor TrkB was mislocalized. Furthermore, also the axonal protein DCC exhibited accumulation coinciding with syntaxin-1a localization. Accumulation of syntaxin-1a, TrkB and CDD was characterized by their presence at the ER which was not due to alterations of post-translational modifications or overexpression of these proteins. Interestingly, the Munc 18-1 mutants displayed misregulated tau phosphorylation, neurofibrillary tangle accumulation and alterations of the ubiquitination state which characterize AD [49]. Moreover, endogenous syntaxin-1a accumulated at the Golgi of Munc 18-1 KO neurons as its export from the Golgi apparatus to the PM and synapsis was altered and cell loss was observed [50]. In support of the fact that syntaxin-1a and SNAP-25 are dysregulated in AD, it has been recently proven that depletion of SNAP-25 induced early Golgi abnormalities and neurodegeneration whereas depletion of syntaxin-1a determined rapid cell death [50]. Nevertheless, the effect on Golgi morphology was not induced by VAMP2 KO [50]. Interestingly, VAMP2 levels correlated with Aβ dimers and pentamers and the abundance of the SNARE protein was reduced when Aβ oligomers were accumulated [40].

Another interesting link between AD and syntaxin-1a is that the SNARE motif of syntaxin-1a interacted specifically with intracellular Aβ monomers and oligomers [51]. Nevertheless, the interaction of the SNARE protein with the oligomers inhibited SNARE complex formation and the opening of the fusion pore [51]. This led to a block in exocytosis, neurotransmission and to cognitive defects [51]. Interestingly, syntaxin-1a has been identified also as an interactor of presenilin 1, a multi-transmembrane protein with a large hydrophilic loop near the C-terminus associated with EOAD, in a yeast two-hybrid assay, however, no functional meaning has been reported so far [52]. Therefore, the SNARE complex composed of SNAP-25, syntaxin-1a and VAMP2 is responsible for regulating some important steps in the onset of AD and of the pathological characteristics of the disease, such as Aβ peptides and tau secretion.

### 2.3. Vamp8 and Secretion of Tau Protein

One of the main causes of AD onset is the phosphorylation of tau protein, its aggregation and intracellular accumulation. Nevertheless, tau protein is also released in the extracellular space and can be found in the CSF. Secretion of tau involves transport from late endosomes to the PM and could help to prevent accumulation of the protein responsible for AD onset [70]. Vamp8 is an R-SNARE that localizes at late endosomes and that can make complexes together with syntaxin-7, vti1b and syntaxin-8 for homotypic fusion of late endosomes, with syntaxin-17 and SNAP-29 for autophagosome-lysosome fusion or with syntaxin-3 and SNAP-23 for secretion [45,77,78].

Interestingly, by overexpressing VAMP8, tau secretion was increased and the amount inside neuroblastoma cells was reduced [70]. In fact, VAMP8-, Rab7- and Rab9-positive vesicles containing tau were directed to the PM where tau was released. Interestingly, a decreased phosphorylation level of tau was detected. Furthermore, overexpression of VAMP8 determined a reduction of the intracellular amount of mutated forms of tau associated with frontotemporal dementia with parkinsonism (FTDP) and alpha-synuclein [70]. It has also been proven that VAMP8 is involved in autophagosome-lysosome fusion [45]. Indeed, presenilin 1, which is the catalytic subunit of the γ-secretase complex, regulated the levels of Aβ. Phosphorylated presenilin 1 at Ser367 site interacted with Annexin A2, which in turn interacted with VAMP8 localized on lysosomes. This SNARE protein facilitated the fusion of lysosomes with autophagosome positive for syntaxin-17 [45]. Interestingly, loss of Annexin A2 decreased VAMP8-syntaxin-17 binding. Finally, the phosphorylation of presenilin 1, interaction with Annexin A2 and formation of the SNARE complex containing VAMP8 and syntaxin-17 led to the progression of autophagy and decrease of Aβ levels due to autophagosome-lysosome fusion [45]. By regulating the fusion of late compartments and the secretion of tau protein VAMP8, together with the other proteins in the SNARE complex, could be involved in the onset and the pathophysiology of AD.

## 3. PD

PD is the second most common neurodegenerative disease and is characterized by the onset of the pathology in adulthood associated with the development of motor symptoms [79]. Familial and sporadic forms of PD have been discovered. However, only a minority of PD cases show mutations in specific genes [80]. Mutations in the *SNCA* gene, which codes for the major component of Lewy bodies named α-synuclein (α-syn), are associated with the disease and genomic variation at this gene is a common PD risk factor. In particular, point mutations and gene duplications or triplications determine the early-onset PD. However, mutations in at least 18 specific chromosomal regions have been linked to the familial form of PD [61,80,81,82,83,84,85,86,87,88]. In some situations of rare familial cases, mutations in the genes called *PARK2* (encoding parkin), *DJ-1* and *PINK1* have been linked to PD and are characterized by a recessive mode of inheritance [89].

PD is normally characterized by the accumulation of misfolded and fibrillary forms of α-syn in surviving neurons, named Lewy Bodies (LBs) and progressive death of dopaminergic neurons in the substantia nigra pars compacta (reviewed in [90]). Interestingly, the degradation of α-syn is dependent on its phosphorylation state and on the functionality of the ubiquitin-proteasome system and autophagy-lysosomal pathway [91]. Furthermore, α-syn is spread outside the cell through extracellular vesicles (EVs) and a high amount is found in the CSF and plasma during the early phase of PD [92]. Moreover, the accumulation of another protein, DJ-1, has been associated with the early onset of PD [92].

It has been hypothesized that neurodegeneration begins with alterations at the synaptic terminals in the striatum and then it progresses along the nigrostriatal pathway causing degeneration of the dopaminergic neurons, therefore PD can be classified as a synapthopathy [55]. Besides synuclein toxicity and oxidative stress also neuroinflammation, mitochondrial dysfunction, lysosomal impairment, endoplasmic reticulum stress, synaptic dysfunction and contribution of glial cells are responsible for apoptosis and progression of the disease [79].

**Figure 2:**
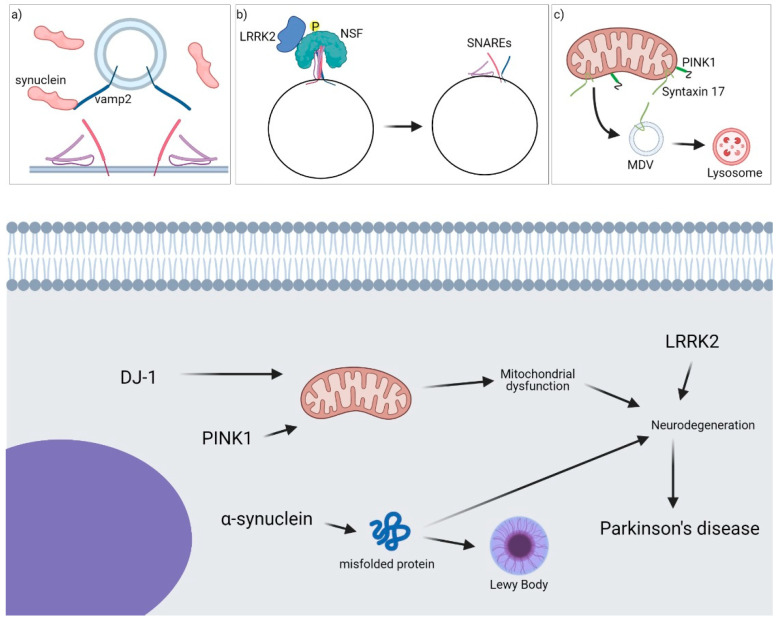
Role of SNARE proteins in PD. A general overview of the main proteins involved in the onset of the pathology is shown. (**a**) α-synuclein binds VAMP2 and inhibits SNARE complex formation at the synaptic site-blocking exocytosis. (**b**) LRRK2 interacts with NSF, phosphorylates it and facilitates SNARE complex disassembly. (**c**) PINK1 and Syntaxin-17 are involved in the formation and fusion of MDVs from mitochondria to endolysosomes/lysosomes.

Under physiological conditions, α-syn can bind directly to SNARE proteins and regulate vesicle trafficking and synaptic transmission (Figure 2) [93]. Moreover, α-syn may play a role in the formation of SNARE complexes by acting as a chaperon [93]. In fact, it has been discovered that α-syn can be in a monomeric cytosolic state or in a multimeric membrane-bound state. Only when it is in the latter state α-syn can act as a chaperone and increase the formation of the complex containing VAMP2, syntaxin-1a and SNAP-25 [53]. Nevertheless, few more SNAREs, such as syntaxin-17 and VAMP4, have been discovered to have also a role in the onset of PD [43,59].

### 3.1. Interaction between α-Syn and SNARE Proteins

One of the roles of SNARE proteins in PD is based on their interaction with one of the key molecules, α-syn, which is involved in the pathogenesis of PD. In fact, it has been demonstrated in vitro that large α-syn oligomers can bind to the N-terminal of Vamp2 [64,65], and proximity ligation assays have proven the co-localization between α-syn and the SNARE complex containing SNAP-25, VAMP2 and syntaxin-1a in cortical primary neurons [47]. Synaptic accumulation of α-syn has been associated with a redistribution of the SNARE proteins. This has been proven in a transgenic mouse line expressing truncated human α-syn(1–120) where symptoms of PD such as aggregates, striatal dopamine deficiency and defects in locomotion, have been discovered [54]. Interestingly, it has been demonstrated that arachidonic acid is able to stimulate the formation of the SNARE complex and subsequently exocytosis through α-syn [48]. In particular, α-syn interacts with VAMP2 and promotes SNARE-dependent vesicle docking. At the contrary, when the phosphatidylserine (PS) is removed from α-syn, it acts instead as an inhibitor for docking [66]. The interaction between α-syn and SNARE proteins can affect synaptic transmission. It has been demonstrated that α-syn interaction with VAMP2 is necessary for α-syn dependent synaptic attenuation [67]. Moreover, it has been proven that two mechanisms regarding α-syn may inhibit SNARE-dependent membrane fusion. Indeed, when non-aggregated α-syn is present at high concentrations vesicle docking is inhibited. However, in the case of lower amounts of α-syn in large oligomers, v-SNARE interaction is impaired [68]. Interestingly, overexpression of the R-SNAREs YKT6 and SEC22b conferred resistance to α-syn inhibition of in vitro assembly of the fusogenic ER/Golgi SNARE complex [46]. Intriguingly, YKT6 is extremely abundant in neurons and in the dopaminergic PC12 cell line where it can be found in specific cytoplasmic particles [94,95]. EVs containing oligomers of α-syn were abundant in peripheral blood from PD patients while syntaxin-1a and VAMP2 levels were reduced [55]. Interestingly, SNAP-25 was increased in CSF of PD patients and different single-nucleotide polymorphisms (SNPs) have been associated with the pathogenesis of PD [61]. Furthermore, a study conducted in mice has shown that in a model of the preclinical stage of PD an increase in the expression of syntaxin-1a was detected whereas in the model of the clinical stage of PD, a reduction in the expression of this gene, and others involved in exocytosis and endocytosis, occurred [41].

Interestingly, α-syn aggregation has been correlated with alterations of the secretory pathway and accumulation of toxic dopamine that occurred in the cytosol. Therefore, impairment of the trafficking between ER and the Golgi apparatus, subsequent Golgi fragmentation and cytoskeleton alterations were related to syntaxin-5 expression [57]. Interestingly, syntaxin-5 is responsible for the formation of extracellular aggregates resembling the amyloid plaques typical of AD [58]. Moreover, over-expression of VAMP8 affected the secretion of α-syn [71,72].

### 3.2. Role of SNAREs in LRRK2-Mediated Synaptic Transmission

Another gene associated with PD is leucine-rich repeat kinase 2 (*LRRK2*), a large kinase protein with GTPase activity, with a role in synaptic vesicle fusion and cytoskeletal-related processes [96]. The mechanism underlying the role of LRRK2 in PD is not fully understood, however, LRRK2 has been discovered to be involved in the process of aggregation and propagation of α-syn [97]. Interestingly, LRRK2 interacts with N-ethylmaleimide sensitive factor (NSF), which is an ATPase involved in the disassembling of the SNARE complex and affects synaptic exocytosis and endocytosis [96]. LRRK2 is able to phosphorylate NSF, thereby facilitating the dissociation of SNARE complexes [96]. The interaction and/or functionality of LRRK2 in the rate of SNARE disassembling is dependent on the phosphorylation of NSF at the threonine 645 site [96]. A yeast two-hybrid screening aiming at identifying LRRK2 functions proved that an interactor of SNAP-25, snapin (SNAP-25-interacting protein), interacted with LRRK2. Moreover, snapin is phosphorylated by LRRK2 on the Thr117, which is relevant for the interaction of the two proteins and for the snapin-SNAP-25 interaction. This protein association has been linked to the exocytosis from hippocampal neurons, thus regulating neurotransmitter release, while protein kinase A (PKA)-mediated phosphorylation of snapin at Ser50 residue affected its binding to SNAP-25 [62]. Furthermore, LRRK2 interacts with VAMP4 and syntaxin-6 during the formation of the SNARE complex including Vti1a and syntaxin-16. Moreover, LRRK2 stabilizes the formation of this SNARE complex with the Golgi-associated retrograde protein (GARP) complex [59]. Furthermore, in a study analyzing the common risk variants of the LRRK2 gene, it has been proved that variations in the VAMP4 gene are associated with the disease [69]. The proven interactions between LRRK2 and the molecules involved in the membrane fusion events demonstrate how new mechanisms could be relevant for the onset of the disease and evaluate new possible targets for novel drugs.

### 3.3. Role of Syntaxin-17 in PINK1/Parkin-Dependent Vesicle Transport Pathway

Parkin is an E3 ligase while PINK1 is a mitochondrially targeted protein kinase. Their role is in the quality control of mitochondria and regulation of the mitochondrial-derived vesicles (MDVs) transport. Impairments of MVs trafficking have been associated with PD pathogenesis as mutations in *PARKIN* and *PINK1* determined recessive forms of PD [98,99,100]. Syntaxin-17 is a peculiar SNARE as it contains two transmembrane domains at the C-terminal region and it has been previously shown to localize at mitochondria [101,102,103]. Syntaxin-17 mediates the fusion of MDVs with endolysosomes facilitating the degradation of damaged mitochondrial content in lysosomes [43,89]. In particular, syntaxin-17 forms a complex together with SNAP-29 and VAMP7 responsible for the delivery of stress-induced MDVs to the late compartments [43]. However, also VAMP8 could make a complex with syntaxin-17 and SNAP-29. Interestingly, syntaxin-17 clustering on the outer mitochondrial membrane was affected by the abundance of parkin or PINK1 as their depletion led to a reduction in the number of syntaxin-17 clusters [43].

## 4. ALS

ALS is an adult-onset neurodegenerative disease characterized by motor system degeneration, progressive muscle atrophy and spasticity followed by respiratory failure and death [104]. Moreover, frontotemporal lobar dementia (FTLD) can occur. Different variants of ALS exist. It is considered familial ALS (fALS) in the case of ALS patients with an ALS-affected family member and the disease is inherited in an autosomal dominant manner. Nevertheless, the majority of cases are sporadic (sALS) [105]. The most common mutation in patients with fALS is in the gene for Cu, Zn superoxide dismutase (SOD1), which determines a toxic gain of function of this cytosolic enzyme that can be followed by misfolded and aggregated SOD1 and cytosolic calcium overload. However, mutations in many other genes (over fifty) have been shown to determine or contribute to the pathogenesis of this neurodegenerative disorder, such as TDP-43, alsin, senataxin, vesicle-associated membrane protein-associated protein B (VAPB), angiogenin, C9orf72, FUS and TARDBP (for more details, refer to [106,107,108,109]). Furthermore, mutations in the neurofilament-heavy subunit, vascular endothelial growth factor (VEGF) and ciliary neurotrophic factor (CNTF) have been associated with ALS [106]. Neuronal death is then determined by oxidative stress, glutamate exitotoxicity, apoptosis, dysregulation of neurotrophic factors and axonal transport, altered endosomal trafficking, impairment of mitochondrial activity, neurofilament dysfunction, alterations in the RNA metabolism, protein misfolding and aggregation, inflammation [106]. The involvement of the glial cells, which surround motor neurons and provide them nutritional and trophic support, makes ALS a multifactorial disorder where several cell types and disease mechanisms induce neuronal death [110].

### VAMP2 Role in Muscle Denervation and Astrocyte-Mediated Toxicity

An important characteristic of neurodegenerative diseases is the presence of protein aggregates containing ubiquitin. Liu and colleagues have evaluated the role of ubiquitination in the functionality of the SNARE complex composed of VAMP2, SNAP-25 and syntaxin-1 in the brain and spinal cord of transgenic mice and how the alteration of this complex due to the modification of VAMP2 with a “noncleavable” N-terminal ubiquitin substrate could lead to adult-onset paralysis and neurodegeneration [30]. This SNARE complex is fundamental for chemical synaptic transmission. Nevertheless, ubiquitination of VAMP2 led to progressive impairment of synaptic transmission at the neuromuscular junction followed by the degeneration of motor nerve terminals [30]. Indeed, an accumulation of tubulovesicular structures at the presynaptic nerve terminal and a reduced synaptic vesicle density were observed while the somata were unaffected by the modified SNARE protein. Interestingly, also synaptic vesicle endocytosis and membrane trafficking were altered when VAMP2 was bound to ubiquitin moiety [30]. Intriguingly, the ubiquitination of syntaxin-1 did not show muscle denervation while the modified form of VAMP2 was still able to interact with syntaxin-1 and SNAP-25 [30]. Therefore, a specific role for VAMP2 in neurodegeneration in ALS has been demonstrated and it is based on altered endocytosis, retention at earlier compartments such as TGN and/or ER and less synaptic transmission. Furthermore, loss of the motor neurons was accompanied by marked astrogliosis [30]. In line with this finding, it has been demonstrated that impaired SNARE-dependent exocytosis contributed to SOD1G93A astrocyte-mediated toxicity in ALS [63]. Astrocytes are ectodermal cells and take part in ion homeostasis, neurotransmitter recycling and metabolic support to the surrounding neurons [111]. Glutamate is the most prominent and main excitatory neurotransmitter. Astrocytes are responsible for the glutamate-glutamine cycle which is important for the synaptic communication between neurons. Indeed, astrocytes internalize glutamate from the synaptic cleft between presynaptic and postsynaptic neurons and avoid excessive postsynaptic stimulation and neuronal death (glutamate mediated excitotoxicity) [110]. Interestingly, astrocytes from the mouse SOD1 mutant model or from human familial and sporadic ALS patients showed a primary role in the pathogenesis of this disease. In fact, astrocytes modulated the release of glutamate, ATP, and D-serine through exocytosis regulated by SNARE proteins [63]. Moreover, intracellular calcium homeostasis is extremely regulated. Elevated intracellular calcium levels are due to its release from the ER after the activation of the inositol 1,4,5-trisphosphate (IP3) receptor. However, a different mechanism of regulating the storage of calcium named store-operated calcium entry (SOCE) is known. SOCE is based on the calcium influx through the plasma membrane calcium channels in response to the depletion of ER calcium. Interestingly, calcium dysregulation in SOD1G93A^+^ astrocytes was due to ER calcium overload and was linked to elevated ATP release which was inhibited by the over-expression of dominant-negative VAMP2. The release of toxic factors was correlated with disease onset [63].

## 5. Conclusions

Degeneration of neurons and other cellular elements to them associated leads to neurodegenerative diseases such as AD, PD, ALS, HD and SMA. One important aspect of these pathologies is the dysregulation of the vesicle trafficking by alterations of the proteins responsible for exocytosis, endocytosis and neuronal survival. On one hand, the death of neuronal cells begins with the distal part of the axon and then the cell body is affected. Impairments of key steps of vesicle exocytosis, such as docking, priming, and Ca^2+^-mediated fusion of vesicles with the presynaptic membrane, are normally responsible for the onset and progression of the disease and SNARE proteins are fundamental components of the machinery that allows membrane fusion and neurotransmitter release at the axon terminal [67,68]. On the other hand, alterations of membrane fusion within the cell body can be connected with protein aggregations and impaired cell functions [43,44,56]. Thirty-six human SNAREs have been discovered and twelve of these have been associated with important steps of intracellular trafficking that are fundamental for cell survival and for avoiding neurodegeneration (Table 1). It is possible to assume that more SNARE proteins could be involved in the regulation of neurotransmitter release or endocytosis of important molecules for neuronal growth or function. Interestingly, few drugs that can be used as medical treatment to relieve the symptoms of AD have Aβ and tau as targets, however, they cannot delay the progression of the disease. In this context, SNAREs could be targeted in order to modulate properly neurotransmitter release and vesicle fusion. Similarly, modulation of SNARE complex formation and activity could be fundamental in order to avoid the intracellular accumulation of specific proteins or the release of neurotoxic molecules.

## Figures and Tables

**Table 1:** Classification of human SNAREs and their involvement in neurodegenerative diseases.

SNARE Type	Name of the SNARE Protein (Alternative Name)	Neurodegenerative Disease	References
Q_a_ (syntaxins)	Syntaxin-1 (HPC-1)	AD, PD, ALS	[30,40,41,47,48,49,50,51,52,53,54,55]
	Syntaxin-2 (Epimorphin)		
	Syntaxin-3		
	Syntaxin-4		
	Syntaxin-5	AD, PD	[42,44,56,57,58]
	Syntaxin-7		
	Syntaxin-11		
	Syntaxin-13 (Syntaxin-12)		
	Syntaxin-16		
	Syntaxin-17	AD, PD	[43,45]
	Syntaxin-18		
Q_b_	GS-27		
	GS-28		
	Vti1a		
	Vti1b		
Q_c_	BET1	AD	[44]
	GS-15		
	Slt1		
	Syntaxin-6	PD	[59]
	Syntaxin-8		
	Syntaxin-10		
Q_b-c_	SNAP-23 (Syndet)		
	SNAP-25	AD; PD; ALS	[30,40,43,47,48,50,53,60,61,62]
	SNAP-29 (GS-32)		
R	VAMP1 (Synaptobrevin 1)		
	VAMP2 (Syb 2)	AD; PD; ALS	[30,40,47,48,53,55,63,64,65,66,67,68]
	VAMP3 (Cellubrevin)		
	VAMP4	PD	[59,69]
	VAMP5		
	VAMP7 (Ti-VAMP)	PD	[43]
	VAMP8 (Endobrevin)	AD, PD	[45,70,71,72]
	YKT6P	PD	[46]
	SEC22b (ERS-24)	PD	[46]
Unclassified	SEC22a		
	SEC22c		
	SEC20 (Bnip1)		

**Table 2:** Molecules affected by the SNARE proteins in the pathophysiology of neurodegenerative diseases.

Neurodegenerative Disease	Molecule Affected	Function
AD	Aβ	Aβ accumulation, secretion, plaque formation
AD	Tau	Cytoplasmic transport, NFTs
AD	Munc 18-1	Regulation of SNARE complex formation, exocytosis and neurotransmission
AD	presenilin 1	Regulation of Aβ production
PD	α-synuclein	Synaptic modulatory protein
PD	LRRK2	Synaptic vesicle fusion
PD	NSF	Regulation of SNARE complex disassembly
ALS	SOD1	Superoxide dismutase
